# Altered Temporal Variations of Functional Connectivity Associated With Surgical Outcomes in Drug-Resistant Temporal Lobe Epilepsy

**DOI:** 10.3389/fnins.2022.840481

**Published:** 2022-04-19

**Authors:** Danni Guo, Li Feng, Zhiquan Yang, Rong Li, Bo Xiao, Shirui Wen, Yangsa Du, Chijun Deng, Xuyang Wang, Dingyang Liu, Fangfang Xie

**Affiliations:** ^1^Department of Neurology, Xiangya Hospital, Central South University, Changsha, China; ^2^National Clinical Research Center for Geriatric Disorders, Xiangya Hospital, Central South University, Changsha, China; ^3^Department of Neurosurgery, Xiangya Hospital, Central South University, Changsha, China; ^4^MOE Key Laboratory for Neuroinformation, High-Field Magnetic Resonance Brain Imaging Key Laboratory of Sichuan Province, School of Life Sciences and Technology, The Clinical Hospital of Chengdu Brain Science Institute, University of Electronic Science and Technology of China, Chengdu, China; ^5^Department of Radiology, Xiangya Hospital, Central South University, Changsha, China

**Keywords:** functional dynamics, surgical outcomes, temporal lobe epilepsy, temporal properties, epilepsy surgery

## Abstract

**Background:**

Currently, more than one-third of patients with drug-resistant temporal lobe epilepsy (TLE) continue to develop seizures after resection surgery. Dynamic functional network connectivity (DFNC) analyses, capturing temporal properties of functional connectivity during MRI acquisition, may help us identify unfavorable surgical outcomes. The purpose of this work was to explore the association of DFNC variations of preoperative resting-state MRI and surgical outcomes in patients with drug-resistant TLE.

**Methods:**

We evaluated 61 patients with TLE matched for age and gender with 51 healthy controls (HC). Patients with TLE were classified as seizure-free (*n* = 39) and not seizure-free (*n* = 16) based on the Engel surgical outcome scale. Six patients were unable to confirm the postoperative status and were not included in the subgroup analysis. The DFNC was calculated using group spatial independent component analysis and the sliding window approach.

**Results:**

Dynamic functional network connectivity analyses suggested two distinct connectivity “States.” The dynamic connectivity state of patients with TLE was different from HC. TLE subgroup analyses showed not seizure-free (NSF) patients spent significantly more time in State II compared to seizure-free (SF) patients and HC. Further, the number of transitions from State II to State I was significantly lower in NSF patients. SF patients had compensatory enhancement of DFNC strengths between default and dorsal attention network, as well as within the default network. While reduced DFNC strengths of within-network and inter-network were both observed in NSF patients, patients with abnormally temporal properties and more extension DFNC strength alterations were less likely to receive seizure freedom.

**Conclusions:**

Our study indicates that DFNC could offer a better understanding of dynamic neural impairment mechanisms of drug-resistant TLE functional network, epileptic brain network reorganization, and provide an additional preoperative evaluation support for surgical treatment of drug-resistant TLE.

## Introduction

Temporal lobe epilepsy (TLE) is a form of focal epilepsy which the function as well as seizures are extremely complicated (Englot et al., 2015). While anti-epileptic drugs can help most patients with TLE become seizure-free, some patients remain with seizures and seek benefit from surgery. In theory, removal or disruption of the ictal onset zone could reduce the abnormal electrical activity and control seizures. Unfortunately, ∼40% of the patients with drug-resistant TLE still suffered seizures after surgery, and the cause remains unclear (Engel et al., 2012). Even the most modern preoperative multidisciplinary clinical evaluations, including seizure semiology, electrophysiology, neuropsychiatric, and imaging assessment, could not virtually distinguish the patients who did not achieve seizure-free after operation from those who completely benefitted from resection surgery.

Several factors are related to favorable post-surgical outcomes for drug-resistant TLE, such as younger age at surgery, shorter disease course, shorter seizure duration, lower seizure frequency, absence of generalized seizures, and the existence of unilateral mesial temporal lobe sclerosis (Specht et al., 1997; Radhakrishnan et al., 1998; Foldvary et al., 2000; Janszky et al., 2005; Ozkara et al., 2008; Muhlhofer et al., 2017). Despite these studies, those factors only had ∼80% chance of predicting surgical outcomes. Therefore, in addition to these clinical and demographic variables, we speculate that there may be other factors that can help distinguish which patient with TLE will obtain favorable surgical outcomes.

Brain network abnormalities are key constituents of TLE disease neuropathology. Functional imaging studies have demonstrated that network reorganization predominantly affects the ipsilateral medial temporal structures as well as the limbic network in TLE (Bonilha et al., 2004, 2010). With a longer disease duration, patients with TLE will experience progressive widespread functional and structural lesions (Galovic et al., 2019). Moreover, decreased functional coupling of the whole-brain network, including the default-mode network, ventral and dorsal attention networks, cognitive networks, as well as increased thalamic “hubness” measured by resting-states functional MRI have been reported in patients with TLE (Fox et al., 2006; Buckner et al., 2008; He et al., 2017). Previous researches indicated that lower network integration globally, whole-network, and within-network connectivity variability had a high prediction accuracy in post-surgical outcomes of patients with TLE, and these studies demonstrated the ability of using resting-state functional MRI network connectivity as a potential clinical tool for surgical result prediction (Morgan et al., 2017; DeSalvo et al., 2020). However, the unfavorable spatial-temporal of functional MRI does not largely consider the presence and potential of temporal variability in understanding the brain functional dynamics. Magnetoencephalography and electroencephalography (EEG) can provide the necessary spatial-temporal information for human brain information processing, but their poor spatial resolution cannot identify the underlying neural degenerative changes.

Dynamic functional network connectivity (DFNC) introduces time-varying characteristics on the basis of functional connectivity (Hutchison et al., 2013). Recent research have explored and identified that capturing these variabilities may engender a new understanding of neuropsychiatric diseases, in particular epilepsy, schizophrenia, autism, Alzheimer’s dementia, and Parkinson’s disease. Overall, the study of dynamic changes can reveal the functional harmony and flexibility of the central nervous system, and the investigation of these transformations could strengthen our understanding of functional diversification and adaptability.

Nonetheless, the whole brain transformation of functional connectivity strength and temporal properties against the background of DFNC remain largely unknown in TLE. The relationship among network reconfiguration, network epileptogenic potential, and clinical phenotype has not been adequately evaluated in drug-resistant TLE. In particular, whether the network configuration is relevant to the surgical outcome remains to be detected. Therefore, the main aim of the present study was to use group independent component analysis, sliding time window approach, and clustering analysis to (i) assess discrepancies in DFNC between patients with TLE and HC and (ii) demonstrate whether surgical outcomes in TLE are associated with altered DFNC temporal properties as well as connectivity strength. We hypothesized that surgical outcomes in drug-resistant TLE are associated with altered DFNC temporal variations, which could possibly be implied as an effective tool for preoperative evaluation.

## Materials and Methods

### Participants

Sixty-one unilateral drug-resistant patients with TLE and 51 age-, gender-matched HC with no history of head trauma or neurological or neuropsychological disease were included in the final analysis. The diagnosis of drug-resistant TLE was determined by the classification of the International League Against Epilepsy (Berg et al., 2010) based on a comprehensive assessment consisting of detailed clinical history, seizure semiology, neurological examination, video EEG monitoring, structural image, and positron emission tomography (PET). In brief, focal aware seizures or impaired awareness; interictal or ictal EEG that shows abnormal discharges over the temporal region; MRI structural and PET metabolism abnormalities in temporal lobe; and seizures cannot be controlled by anti-epileptic drugs. Inclusion criteria included the diagnosis of TLE, the diagnosis of drug-resistant epilepsy, and no contraindications for surgical resection. Exclusion criteria included pre-surgical intracranial monitoring, progressive neurological disease, focal lesion aside from temporal region, and combination with severe mental disorders. Only 59 of 61 patients with drug-resistance TLE underwent anterior temporal lobectomy (*n* = 43), selective amygdalohippocampectomy (*n* = 7), or temporal lobe lumpectomy (*n* = 9) at the Department of Functional Neurosurgery, Xiangya Hospital of Central South University from 2018 to 2020. Two patients decided not to undergo surgery after the preoperative evaluation, and we lost contacts with four post-operative patients during the follow-up period. Therefore, we only included 55 patients with TLE in the further subgroup analysis. There were a total number of 36 SF patients and 19 NSF patients. Seizure outcomes of the 55 patients were assessed by an epileptologist at each year post surgery (up to 2 years) using the Engel surgery outcome classification (Engel et al., 2003) as seizure-free (SF; Engel Class I) and not seizure-free (NSF; Engel Class II through IV).

This study was approved by the Ethics Committee of Xiangya Hospital, and all participants provided written informed consent according to the Declaration of Helsinki. The demographic and clinical information of all participants are presented in Table 1.

**TABLE 1:** Demographic and clinical characteristics from study groups.

Variables	HC	SF	NSF	*P* value		
	(*n* = 51)	(*n* = 39)	(*n* = 16)	HC vs SF	HC vs NSF	SF vs NSF
Age, years, mean (range)	30.7 (18–56)	28.1 (14–55)	33.3 (17–56)	0.34	0.37	0.07
Sex (female/male)	19/32	18/21	7/9	0.40	0.64	0.87
Handedness (right/left)	51/0	39/0	16/0	> 0.99	>0.99	> 0.99
Age at onset, years, mean (range)	–	14.1 (1–32)	15.5 (1–45)	–	–	0.69
Epilepsy duration, years, mean (range)	–	14.0 (1–32)	17.8 (3–37)	–	–	0.16
Lesion side (right/left)	–	18/21	11/5	–	–	0.13
All Seizures Frequency, per month, mean (range)	–	9.9 (1–33)	11.7 (3–45)	–	–	0.49
GTCS Frequency, per month, mean (range)	–	1.3 (0–13)	1.3 (0–5)	–	–	0.99
Status Epilepticus	–	*n* = 2	*n* = 3	–	–	0.11
Number Of Meds Failed, mean (range)	–	4.3 (2–8)	4.2 (2–7)	–	–	0.79
MTS On Pathology	–	28	13	–	–	0.46
Interictal EEG (Lat/Not Lat)	–	39/0	12/4	–	–	0.0012
Ictal EEG (Loc/Not Loc/No Records)	–	16/4/19	7/0/7	–	–	0.44
Follow-up, months (range)	–	18.2 (12–24)	18.8 (12–24)	–	–	0.74
Type Of Surgery (ATL/SelAH/TLL)	–	28/5/6	11/2/3	–	–	0.95

*Values are given as mean (range). Statistical differences are listed between HC and SF (HC vs SF), HC and NSF (HC vs NSF), and SF and NSF (SF vs NSF). Chi-square test was used for categorical variables. ATL, anterior temporal lobectomy; EEG, electroencephalography; GTCS, generalized tonic clonic seizures; HC, healthy controls; Lat, lateralized; Loc, localized; Meds, anti-epileptic medications; MTS, mesial temporal sclerosis; NSF, not seizure-free; SelAH, selective amygdalohippocampectomy; SF, seizure-free; TLL, temporal lobe lumpectomy.*

### MRI Acquisition

Preoperative resting-state fMRI data for all patients with TLE and HC were acquired on a 3.0 Tesla Siemens Prisma MRI system with a standard 32-channel head coil (Xiangya Hospital of Central South University). During the MRI scanning, all participants were instructed to keep the head steady, eyes closed without falling asleep, and relax without particular thinking. Scans were scanned using echo planar imaging sequences set to the following parameters: TR = 720 ms, TE = 37 ms, flip angle = 52°, 64 axial slices with 2.5 mm thickness and 2.5-mm gap, matrix size = 90 × 90, field of view = 225 mm × 255 mm, voxel size = 2.5 mm × 2.5 mm × 2.5 mm. Each resting-state functional sequence lasted 9.456 min, resulting in 788 volumes.

### Controlling for Head Motion

We applied stringent control criteria to reduce the potential head movement bias (Hutchison et al., 2013). Specifically, we calculated maximum displacement and mean frame-wise displacement. Eventually, we excluded participants with excessive head movement (maximum displacement value over 0.3 mm or mean frame-wise displacement value exceeding 3 mm) during the scan.

### Resting-State Functional MRI Data Preprocessing

Resting-state functional MRI data were preprocessed using Data Processing Assistant DPARSF (V4.3),^1^ which is based on Statistical Parametric Mapping software package SPM 12^2^ implemented in MATLAB (version R2018b, MathWorks, Inc., Natick, MA, United States). The first 18 scans were discarded to achieve magnetization equilibrium, resulting in a total of 770 volumes. Slice timing was used to correct the slice acquisition delay, spatial realignment was performed for motion correction, images were normalized to Montreal Neurological Institute (Engel et al., 2003) space using the standard EPI template and interpolated to 3 mm cubic voxel, and spatial smoothing was applied with 6 mm full width at half maximum (FWHM) Gaussian kernel.

### Group Independent Component Analysis

After resting-state functional MRI data preprocessing, intrinsic connectivity networks of all subjects were created. We implemented group independent component analysis in GIFT within the functional MRI Toolbox (GIFT version 3.01).^3^ Group independent components (ICs) were obtained by concatenating the preprocessed resting-state data from all participants. During the principal component analysis, two data reduction steps were performed, including subject-specific and group-level steps. Using the principal component analysis, the subject-specific data were reduced to 120 principal components. Further, in the group-level data reduction, the concatenated data were reduced to 100 group ICs with the expectation maximization algorithm (Roweis, 1999). The reliability and stability of the ICA algorithm were performed by repeating it 30 times using ICASSO in GIFT (Himberg et al., 2004). The obtained ICs with within-cluster similarity values greater than 0.80 were selected to estimate their reliability and stability. Subject-specific time and spatial maps for each IC were created using the back-reconstruction algorithm (Calhoun et al., 2001).

Among the obtained 100 ICs, the spatial map should exhibit peak activation in the grey matter, low spatial overlap with susceptibility artifacts of cerebral vessels as well as ventricles, and time courses that were mainly of low-frequency fluctuations with a power ratio of 0.15–0.25 Hz (Cordes et al., 2000). According to these criteria, we identified 33 meaningful ICs, and they were classified into nine instinct connectivity networks, based on the spatial correlation values between each IC and network templates (Yeo et al., 2011; Shirer et al., 2012; Szaflarski et al., 2018). ICs are arranged into cerebellum (CB), dorsal attention network (DAN), default mode network (DMN), frontoparietal network (FPN), limbic (LIM), subcutaneous (SC), sensorimotor network (SMN), ventral attention network (VAN), and visual network (VN).

To reduce the detrend linear, quadratic, and cubic trends, additional post-processing was performed for the time cours of 33 ICs. Outliers were detected based on the 3DDESPIKE algorithm.^4^ The fifth-order Butterworth filter with a high-frequency cut-off of 0.15 Hz was selected for filtering processing. Finally, movement parameters were regressed out.

### DFNC Analysis

#### Sliding Time Window Approach

Dynamic functional network connectivity analyses were investigated with the sliding window approach in GIFT. Resting-state time series data were split into windows of the size of 60 repetition times (∼44 s), convolving a rectangle with a Gaussian and sliding a step with one repetition time. Previous studies have shown that cognitive states could be identified within a window length of 30–60 s, while the topological transformation of the brain network began to stabilize at 30 s (Shirer et al., 2012). Our window length has been testified to provide a good balance between the precision of covariance matrix estimation and the ability of DFNC calculation. Since short time series may have not enough information to represent the full covariance matrix, the inverse covariance matrix was used to estimate covariance in this study (Smith et al., 2011). Further, following the graphic LASSO method, we placed additional 100 repetitions on the L1 norm of the accuracy matrix to advance sparsity (Friedman et al., 2008). To stabilize variance prior to further analysis, Fisher’s z-transformation was used to transform DFNC matrices to z-scores.

#### Clustering Analysis

Window functional connectivity correlation matrix was calculated using K-means clustering algorithm to obtain the frequency and structure of reoccurring functional connectivity states (Roweis, 1999; Lloyd, 2006). L1 distance (City distance) function, as an effective measure for high-dimensional data, was used for K-means clustering algorithm (Aggarwal et al., 2001). Furthermore, to estimate the optimal number of clusters for group clustering, a cluster number validity analysis was performed on the exemplar windows of all subjects by varying *k* from 2 to 10. According to the gap criterion of cluster validity index, under a null distribution of a reference, the standardized within-cluster dispersion was expected to merge with within-cluster sum of squares, as well as the silhouette criterion, the similarity between points in other clusters and windows in the same cluster (Peter, 1987; Tibshirani et al., 2001). Given these two predictive criteria, we finally determined that the optimal cluster number was two (*k* = 2).

#### Group Differences in DFNC

We evaluated the following temporal properties: (i) fractional windows (the percentage of total time spent by subjects in a given state); (ii) mean dwell time (the time subjects spent in one state without switching to another state); and (iii) number of transitions (the frequency of subjects changing their state). We also tested for differences between groups in DFNC pairs for each connectivity state. Connectivity strength computation of all connectivity pairs in each state (528 pairings; *P* < 0.05, FDR-corrected) between TLE and HC, and among SF, NSF, and HC were both based on templates available in the GIFT toolbox implemented in MATLAB.

### Statistical Analysis

All statistical analyses were performed using SPSS version 22 (IBM Corporation, Armonk, NY, United States). The differences between TLE and HC were tested using a two-sample independent *t*-test, while between-group differences among the TLE subgroups (SF and NSF) and HC were investigated using the three-level one-way ANOVA. *Post hoc t*-tests were added in case of significant ANOVA results. Pearson’s Chi-square test was used to compare the categorical variables. The level of significance was *p* < 0.05 (two-sided significant testing), and multiple comparisons were performed using the false discovery rate (FDR)-corrected.

## Results

### Demographic and Clinical Characteristics

Sixty-one patients with drug-resistant TLE and 51 HC were included in the whole analysis. There were no significant differences in age and gender. There were a total number of 36 SF patients and 19 NSF patients involved in further subgroup analysis. There were no significant differences between SF and NSF patients under each clinical variable except the interictal EEG (Table 1).

### Intrinsic Functional Connectivity Networks

**FIGURE 1:**
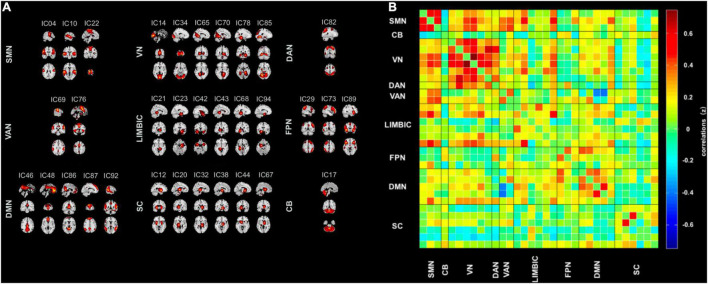
Spatial maps of the 33 intrinsic connectivity networks and the stationary functional connectivity. **(A)** Independent component networks spatial maps divided into nine functional networks: cerebellum (CB, one component), dorsal attention network (DAN, one component), default mode network (DMN, five components), frontoparietal network (FPN, three components), limbic (LIM, six components), subcutaneous (SC, six components), sensorimotor network (SMN, three components), ventral attention network (VAN, two components), and visual network (VN, six components), based on their anatomical and functional properties. **(B)** Group averaged static functional network connectivity between independent component pairs computed by the rest state functional MRI data. Connectivity values in the correlation matrix represents the Fisher’s z-transformed Pearson correlation coefficient, averaged over all subjects. IC, independent component.

Thirty-three ICs were divided into the following nine networks: CB (IC 7), DAN (IC 82), DMN (IC 7), FPN (ICs 29, 73, 89), LIM (ICs 21, 23, 42, 43, 68, 94), SC (ICs 12, 20, 32, 38, 44, 67), SMN (ICs 4, 10, 22), VAN (ICs 69, 76), and VN (ICs 14, 34, 65, 70, 78, 85). Figure 1A displays the detailed information and spatial maps of ICs. Figure 1B shows the averaged intrinsic functional network connectivity between 33 ICs for 61 patients with TLE as well as 51 HC.

### DFNC Analysis

#### Clustering Analysis

**FIGURE 2:**
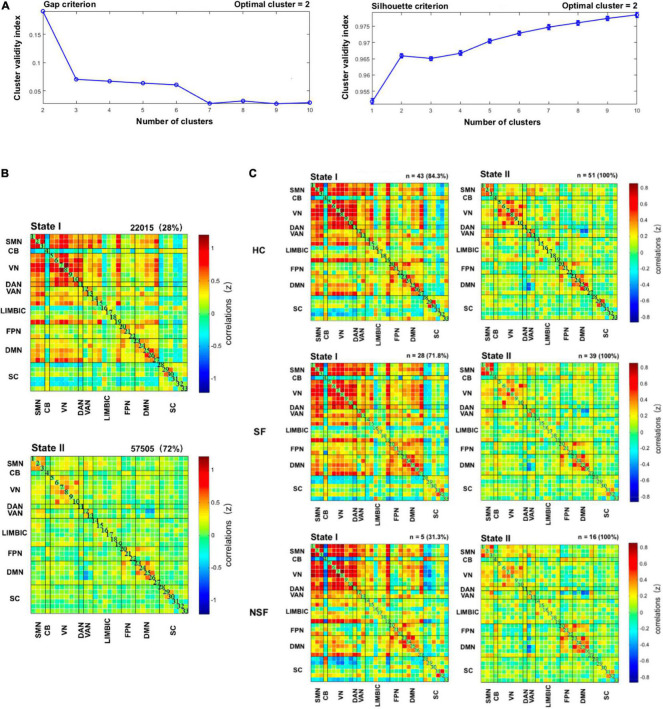
Results of clustering analysis. **(A)** Gap criterion: the optimal number of clusters as equal to two (*k* = 2); Silhouette criterion: the optimal number of clusters equal to 2 (*k* = 2). The clustering algorithm was applied to all participants. **(B)** Cluster medians matrices for each state. The total number and percentage of occurrences are listed upper each cluster right corner. **(C)** Dynamic functional network connectivity states for each of the three subgroups. CB, cerebellum; DAN, dorsal attention network; DMN, default mode network; FPN, frontoparietal network; HC, healthy controls; LIM, limbic; NSF, not seizure-free; SC, subcutaneous; SF, seizure-free; SMN, sensorimotor network; VAN, ventral attention network; VN, visual network.

The optimal criteria for the number of states is shown in Figure 2A. Given these two predictive criterion, the optimal cluster number was determined to the value 2 (*k* = 2). Figure 2B displays these two functional network connectivity states and their visualized connectivity patterns. We identified two completely distinct functional network connectivity states. As noted in Figure 2B, State I of patients with drug-resistant TLE and HC is a less frequent brain state (overall frequency: 28%, Figure 2B, upper panel), but it has strong positive inter-network connectivity, located mainly between SMN, VN, DAN, and VAN and State II is a more frequent brain state (overall frequency: 72%, Figure 2B, below panel) with within-network connectivity dominating, located mainly within SMN, VN, and DMN. Figure 2C shows the group-specific *k*-means algorithm results, and as noted above, there were 43 HC (percentage: 84.3%, Figure 2C, upper panel), 28 SF patients (percentage: 71.8%, Figure 2C, middle panel), and 5 NSF patients (percentage: 31.3%, Figure 2C, below panel) who entered State I.

#### DFNC Strength

**FIGURE 3:**
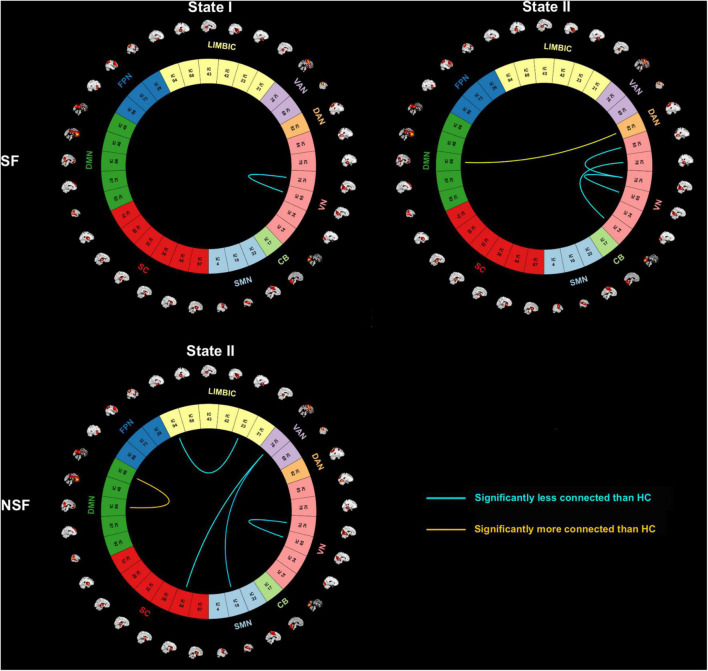
Dynamic functional connectivity state results. Subgroup-specific circle plots of significant dynamic functional connectivity differences in each state, where SF and NSF patients had a weaker or stronger connectivity pattern in comparison to the HC. (post hoc t-tests, *P* < 0.05, FDR-corrected for multiple comparisons). CB, cerebellum; DAN, dorsal attention network; DMN, default mode network; FPN, frontoparietal network; HC, healthy controls; LIM, limbic; NSF, not seizure-free; SC, subcutaneous; SF, seizure-free; SMN, sensorimotor network; VAN, ventral attention network; VN, visual network.

In State I, within-network connectivity of VN was weaker in patients with TLE compared to HC (three-level ANOVA: *P* < 0.05, *post hoc* t-test: *P* < 0.05, FDR-corrected). In State II, a stronger inter-network connectivity (DMN-DAN) was observed in SF patients, as well as a stronger within-network connectivity (DMN-DMN) was observed in NSF patients, compared to HC. While SF patients had a weaker connection in VN-VN, NSF had weaker connections in LIM-LIM, VAN-SC, VAN-SMN, and VN-VN (three-level ANOVA: *P* < 0.05, *post hoc t*-test: *P* < 0.05, FDR-corrected) (Figure 3).

#### Temporal Properties

**FIGURE 4:**
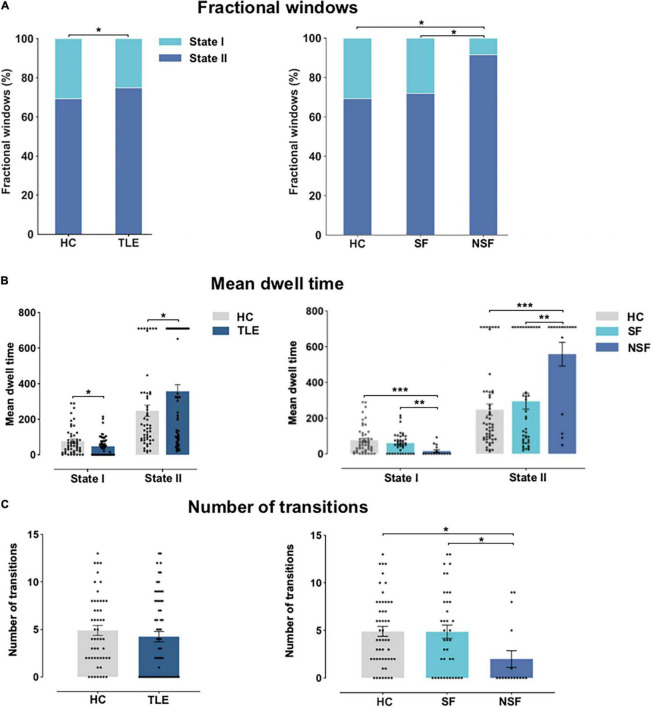
Temporal properties of DFNC for TLE and HC, and across TLE surgical outcome subgroups. **(A)** Fractional windows: Percentage of total time subjects spent in each state. **(B)** Mean dwell time and **(C)** number of transitions between states. Asterisks indicate statistically significant group differences based on significant post hoc t-tests (*P* < 0.05, FDR-corrected). HC, healthy controls; NSF, not seizure-free; SF, seizure-free; TLE, temporal lobe epilepsy. *P < 0.05; **P < 0.01; ***P < 0.001.

Figure 4 shows temporal properties of DFNC for TLE and HC groups, as well as SF patients, NSF patients, and HC. In TLE, State II was more frequently observed than State I (*P* = 0.039), whereas in HC, State II occurred less frequently and State I more commonly (*P* = 0.039) compared to patients with TLE. Further, State II was more frequent in NSF than in SF and HC (NSF-SF: *P* < 0.05, NSF-HC: *P* < 0.05, FDR-corrected), while the opposite pattern was observed in State I, which was less frequent in NSF than in SF and HC (NSF-SF: *P* < 0.05, NSF-HC: *P* < 0.05, FDR-corrected) (Figure 4A).

As exhibited in Figure 4B, significant group differences in mean dwell time between TLE and HC were identified in State I (*P* < 0.05) and State II (*P* < 0.05). Subgroup analysis revealed that NSF patients spent significantly less time than SF patients and HC in State I (NSF-SF: *P* < 0.001, NSF-HC: *P* < 0.001, FDR-corrected). NSF patients spent more time in State II as compared to SF patients and HC (NSF-SF: *P* < 0.01, NSF-HC: *P* < 0.001, FDR-corrected). Patients with TLE changed less frequently between these two states than HC (*P* = 0.52). Finally, subgroup analysis indicated that NSF group performed fewer transitions than SF group and HC (NSF-SF: *P* < 0.05, NSF-HC: *P* < 0.05, FDR-corrected) (Figure 4C). Overall, these DFNC changes suggested that patients with TLE with unsatisfied post-surgical outcomes stay longer in the sparse within-network connectivity state, performed fewer transitions, and had shorter dwelling time in the strong inter-network connectivity state.

Figures 1–3 were created using GIFT toolbox (v3.01). Figure 4 was made using Graph Pad Prism 8.0 software. MATLAB scripts for DFNC computation were based on templates available in the GIFT toolbox.

## Discussion

In this present study, we sought to predict post-surgical outcomes based on data derived from the pre-surgical evaluation of patients with drug-resistant TLE. We innovatively applied the DFNC analysis to evaluate the differences between 61 patients with drug-resistant TLE and 51 HC, ranging from favorable and unfavorable surgical outcomes of patients with TLE, putting a particular emphasis on temporal properties (fractional windows, mean dwelling time, and number of transitions), and the brain network connection strength of distinct connectivity states. We identified two different dynamic connectivity states, which were significantly related to surgical outcomes. The most altered temporal variations were observed in NSF patients. Network connection changes were apparent in VN-VN, DMN-DAN, DMN-DMN, LIM-LIM, VAN-SC, and VAN-SMN. In summary, this is the first study to assess dynamic connectivity properties across the TLE surgical outcomes. We believe that DFNC analyses permit the evaluation of time-varying characteristics on the basis of functional connectivity, may reflect the more comprehensive functional capacity of the central nervous system, and thus, it may serve as a potential clinical imaging biomarker of the disease (Deco et al., 2011; Hutchison et al., 2013; Kucyi et al., 2017).

Importantly, we have shown that temporal properties (fractional windows, mean dwelling time, and number of transitions) are altered in patients TLE vs. HC. Furthermore, in patients with poor prognosis, these changes were especially notable. We observed weaker connections in SF and NSF within the VN compared to HC which may indicate that patients with TLE had poor visual information processing ability. However, the mechanism of visual function impairment in TLE is currently not well understood. Previous studies of saccadic eye movements have found a connection between hippocampal activity and visual exploration in rodents (Jutras et al., 2013). Our study had over 70% of the enrolled patients with postoperative pathology confirming unilateral hippocampal sclerosis. A latest study found that the hippocampus may not be limited to a single-working mode of visual memory storage. It may also integrate visual information and interact with the frontoparietal eye movement region to assist in visual exploration, thus improving the efficiency of information acquisition (Bridge et al., 2017). Despite the similarity of our results with previous studies, further investigations of the mechanism on drug-resistant TLE with hippocampal sclerosis-related VN deficits are needed.

Then, we found a stronger connection between the DMN-DAN in SF, and NSF had a stronger connection within the DMN in the segregated state compared to HC. DAN and DMN were distinct functional networks and these two networks operate in an opposite pattern in the human brain (Fox et al., 2005). Besides, the DMN of the epileptic brain was thought to be more likely to transition between states than the healthy brain (Yang et al., 2021). It may suggest that patients with TLE had an overresponse during the dynamic balance of DMN. Our results further confirmed the important position of DMN in TLE networks. Our results have some consistency with previous studies, but there are also have some differences. This condition makes us have the reason to believe that our further disease subgroup analysis in this study could help us to better understand the significance of dynamic changes in the functional network connectivity. We hypothesized that enhanced DMN-DAN connectivity strength in SF patients is a compensatory mechanism that will disappear as the brain network further disrupts. The existence of the dynamic functional connectivity compensation mechanism of preoperative brain networks in patients may predict a better surgical outcome, but this still needs to be confirmed by further studies.

In addition, we detected that NSF had weaker connections in LIM-LIM, VAN-SC, VAN-SMN in State II compared to HC. The interaction of attention-sensorimotor network is important for movement control and skill learning. Unfortunately, patients with TLE suffered from impaired multiple functions due to chronic recurrent seizures (Glickstein, 2000). Coordinating functions of activity within-network and between-network were closely interrelated with key interacting functional networks (Greicius et al., 2003; Fox and Raichle, 2007). Chronic seizures induced a pervasive disturbance of network behavior that may influence the consistency of functional and effective connectivity and lead to the decline of brain overall function (Friston, 2011; Jiang et al., 2018). Previous studies showed that patients with focal epilepsy demonstrated extensive network alterations, including the functional and structural networks’ abnormal intergration (van Diessen et al., 2014). DeSalvo et al. (2020) found that lower overall network integration of preoperative resting-state functional MRI scans was associated with persistent postoperative seizures in patients with TLE. In general, these results explained that the different surgical outcomes detected in TLE may be related to the observed alterations of more extensive within-network and inter-network.

Further, we observed that the overall frequency of State II in patients with TLE occurred more often than in HC, and along with it the appearance of State I was lower. Our results concur with the DFNC research by Liao et al. (2014) who revealed dynamics of functional connectivity, adaptive reconfiguration of functional brain networks, and confirmed the vulnerability of the resting-state functional network in epilepsy. The network reconstruction of patients with TLE is more inclined to increase the intra-network connection and decrease the connections between networks. Moreover, patients with TLE diverged from HC depending on their surgical outcomes. We detected differences among SF, NSF, and HC. This characteristic network reconstruction model was more obvious in NSF patients. Further. post hoc analysis showed that State II was observed more frequently in NSF patients than in SF and HC groups; whereas there was no significant difference between SF patients and HC.

Finally, we found that there were significant differences in DFNC temporal properties between patients with TLE and HC. These diverse temporal properties were particularly evident in NSF patients, who spent the longest time in State II and remained for a minimum amount of time in the strongly inter-network connection State I than SF. There were consistent pieces of evidence which indicated that weak functional connectivity between networks, along with relative increases in functional connectivity within networks, were interpreted as the reduced integration efficiency of the neural network associated with disease expression (Chan et al., 2014; Elman et al., 2016). Moreover, active inter-state transition indicated better functional flexibility (Nomi et al., 2017). Our results implied that patients with TLE, in particular, NSF patients had the inefficient and unstable information flow within/between functional networks as well as the abnormal integration of brain networks. Our results are consistent with the findings in other neurological disorders, such as Alzheimer’s disease, Parkinson’s disease, and schizophrenia, all showing abnormal temporal properties compared to HC (Damaraju et al., 2014; Liu et al., 2017; Lottman et al., 2017; Diez-Cirarda et al., 2018). Aligned with these reports, our observations highly suggested that the temporal properties of functional network connectivity were closely related to surgical outcomes of TLE. Logically, patients with TLE with abnormally temporal variations were less likely to achieve seizure freedom.

There are several limitations of our study that require discussion. First, the small sample size impedes accurate estimates of generalizability. However, our sample is designed to maximize homogeneity to understand a specific population of patients. The proposed relationships with outcomes, therefore, need validation in a larger, independent patient cohort. Second, our data only include a 2-year follow-up and are measured only in yearly increments. More detailed dates of recurrence would improve future studies. Third, our cohort included patients who underwent three different types of surgical treatment. While no difference was detected in the outcome between selective amygdalohippocampectomy vs. anterior lobectomy in a recent study, this should be considered in future validation studies. Finally, we realize that there is a methodological limitation in this study. As none of the 61 patients underwent postoperative MRI scans, the exact amount of excision tissue in these patients could not be measured. However, our research objective was to explore preoperative changes related to surgical outcomes, which could help to guide preoperative evaluation.

## Conclusion

In summary, this is the first study to evaluate DFNC properties resulting in the TLE surgical outcomes. Significantly, we have demonstrated altered temporal properties (fractional windows, mean dwelling time, and number of transitions) in patients with TLE as compared to HC. Furthermore, in patients with poor prognosis, these changes were especially notable. Specifically, NSF patients have more extensive alterations in DFNC strength within and between networks. Patients with TLE with preoperative abnormal temporal variations were less likely to achieve postoperative seizure freedom. We argue that this research approach, in particular the temporal properties of DFNC, could be a useful imaging biomarker for predicting surgical outcomes in TLE.

## Data Availability Statement

The original contributions presented in the study are included in the article/Supplementary Material, further inquiries can be directed to the corresponding authors.

## Ethics Statement

The studies involving human participants were reviewed and approved by the Ethics Committee of Xiangya Hospital. Written informed consent to participate in this study was provided by the participants’ legal guardian/next of kin.

## Author Contributions

DG conceived designed, and initialized the study, analyzed the data, organized the figures, and wrote the manuscript. FX managed all parts of the experimental Resting-state fMRI data acquisitions. ZY and DL performed preoperative evaluations as well as surgical operations of drug-resistance TLE patients. LF, DL, ZY, and BX diagnosed the drug-resistance TLE patients and rigorously determined the experimental enrolled patients. RL, CD, and XW assisted with data analysis. LF and RL designed experiments and edited the manuscript. SW and YD assisted with collection and collation of clinical data of TLE patients and managed the follow-up of postoperative patients. All authors contributed to the article and approved the submitted version.

## Conflict of Interest

The authors declare that the research was conducted in the absence of any commercial or financial relationships that could be construed as a potential conflict of interest.

## Publisher’s Note

All claims expressed in this article are solely those of the authors and do not necessarily represent those of their affiliated organizations, or those of the publisher, the editors and the reviewers. Any product that may be evaluated in this article, or claim that may be made by its manufacturer, is not guaranteed or endorsed by the publisher.

## Footnotes

1
http://www.rfmri.org/DPARSF

2
www.fil.ion.ucl.ac.uk/spm

3
http://icatb.sourceforge.net

4
http://afni.nimh.nih.gov/afni
