# Supplementary material for: Altered Temporal Variations of Functional Connectivity Associated With Surgical Outcomes in Drug-Resistant Temporal Lobe Epilepsy
Source: Front Neurosci. 2022 Apr 19;16:840481. doi: 10.3389/fnins.2022.840481 (PMC9063407; doi:10.3389/fnins.2022.840481)
Supplement: Supplementary file 1 [file Data_Sheet_1.PDF]

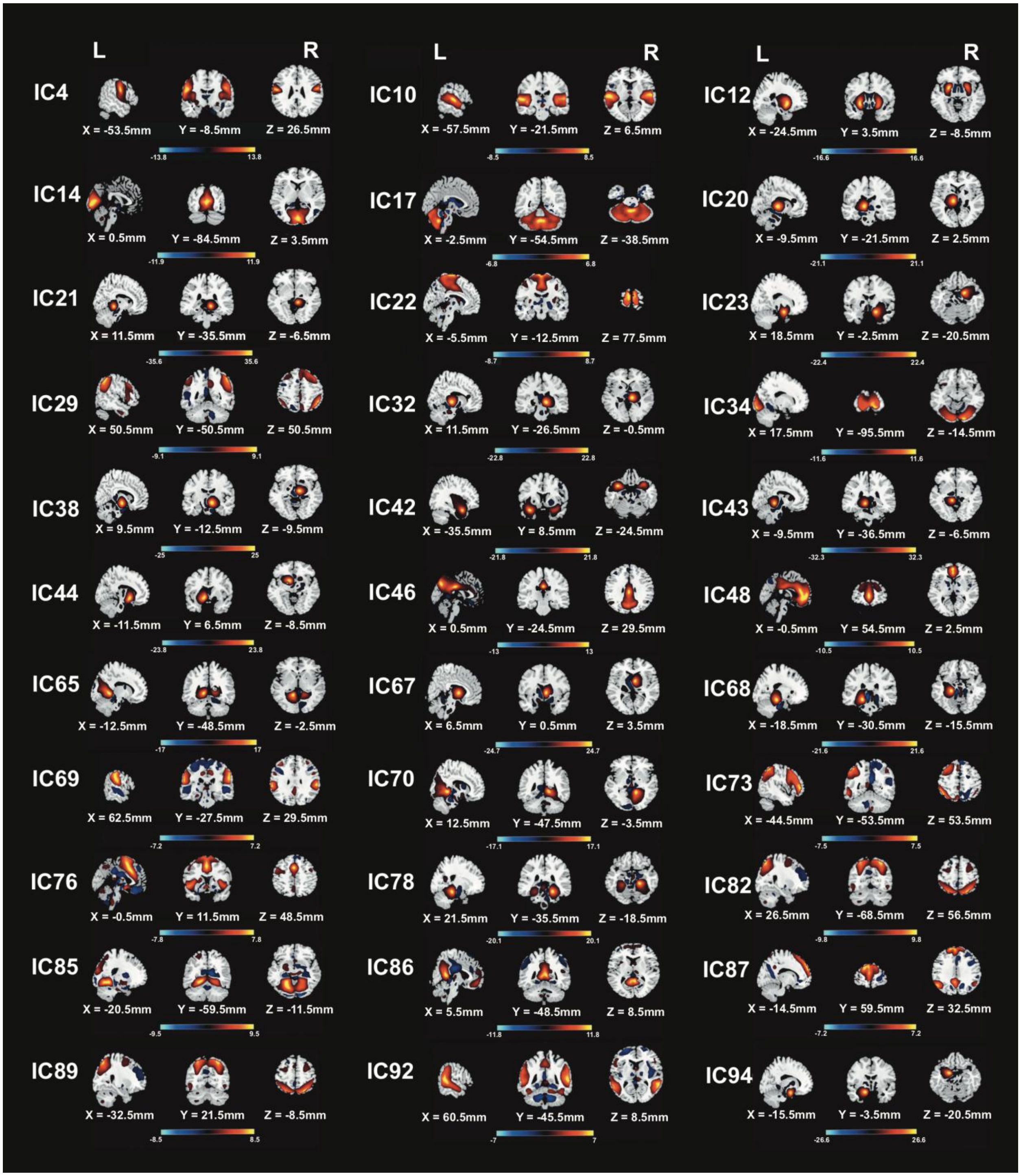

Supplementary Figure 1 Spatial maps of the 33 intrinsic connectivity networks and peak coordinates.

The color bar represents t-values.

IC = independent component; L = left; R = right.
